# Clam focal and systemic immune responses to QPX infection revealed by RNA-seq technology

**DOI:** 10.1186/s12864-016-2493-9

**Published:** 2016-02-27

**Authors:** Kailai Wang, Carmelo del Castillo, Erwan Corre, Emmanuelle Pales Espinosa, Bassem Allam

**Affiliations:** School of Marine and Atmospheric Sciences, Stony Brook University, Stony Brook, NY 11794-5000 USA; Analyses and Bioinformatics for Marine Science, Station Biologique de Roscoff, 29688 Roscoff Cedex, France

**Keywords:** Hard clam, *Mercenaria mercenaria*, QPX, RNAseq, Immune response, Focal, Systemic

## Abstract

**Background:**

The hard clam *Mercenaria mercenaria* is an important seafood species widely exploited along the eastern coasts of the United States and play a crucial role in coastal ecology and economy. Severe hard clam mortalities have been associated with the protistan parasite QPX (Quahog Parasite Unknown). QPX infection establishes in pallial organs with the lesions typically characterized as nodules, which represent inflammatory masses formed by hemocyte infiltration and encapsulation of parasites. QPX infection is known to induce host changes on both the whole-organism level and at specific lesion areas, which imply systemic and focal defense responses, respectively. However, little is known about the molecular mechanisms underlying these alterations.

**Results:**

RNA-seq was performed using Illumina Hiseq 2000 (641 Million 100 bp reads) to characterize *M. mercenaria* focal and systemic immune responses to QPX. Transcripts were assembled and the expression levels were compared between nodule and healthy tissues from infected clams, and between these and tissues from healthy clams. *De novo* assembly reconstructed a consensus transcriptome of 62,980 sequences that was functionally-annotated. A total of 3,131 transcripts were identified as differentially expressed in different tissues. Results allowed the identification of host immune factors implicated in the systemic and focal responses against QPX and unraveled the pathways involved in parasite neutralization. Among transcripts significantly modulated upon host-pathogen interactions, those involved in non-self recognition, signal transduction and defense response were over-represented. Alterations in pathways regulating hemocyte focal adhesion, migration and apoptosis were also demonstrated.

**Conclusions:**

Our study is the first attempt to thoroughly characterize *M. mercenaria* transcriptome and identify molecular features associated with QPX infection. It is also one of the first studies contrasting focal and systemic responses to infections in invertebrates using high-throughput sequencing. Results identified the molecular signatures of clam systemic and focal defense responses, to collectively mediate immune processes such as hemocyte recruitment and local inflammation. These investigations improve our understanding of bivalve immunity and provide molecular targets for probing the biological bases of clam resistance towards QPX.

**Electronic supplementary material:**

The online version of this article (doi:10.1186/s12864-016-2493-9) contains supplementary material, which is available to authorized users.

## Background

The hard clam, *Mercenaria mercenaria*, is an ecologically- and economically-important marine bivalve species that thrives along the northeastern coasts of the United States and Maritime Canada. In the past few decades, the hard clam industry has been severely impacted by a protistan parasite called QPX (Quahog Parasite Unknown), which is responsible for mortality episodes in both wild and cultured clam populations [1–7]. QPX is believed to be an opportunistic pathogen and has been detected in a wide variety of environmental substrates including sediments, marine aggregates and other organic matrices [8–10]. Interestingly, previous reports highlight the ability of QPX to sustain very low abundance in clams without causing disease outbreaks until it encounters hosts with reduced immunity or following shifts of environmental conditions that add to the virulence of the parasite, under which conditions QPX can take advantage to establish infection sometimes leading to severe clam mortality events [4, 11].

Lesions caused by QPX, usually associated with the presence of nodules, are commonly found in pallial tissues, such as alongside the inner rim of the mantle or at the base of the siphon [1, 2]. These places are widely considered as the portal of entry for QPX cells acquired from the surrounding environment during suspension-feeding [5, 7]. The QPX nodules represent inflammatory masses containing both parasite cells and abundant clam hemocytes, resulting from a series of comprehensive host immune responses induced by the infection that leads to massive focal hemocyte infiltration, parasite encapsulation and partial necrosis of the affected area [1]. Like other invertebrates, the hard clam lacks the specific immune responses and their defense mechanism mainly relies on the effectors of innate immunity, which is mediated by circulating hemocytes and highly diversified humoral antimicrobial factors. These cellular and humoral immune components work in a synergistic way to initiate the recognition, segregation and ultimately elimination of pathogens and other non-self entities [12, 13]. The launching of innate immune responses involves myriad cellular and humoral events modulated not only at the infection sites (focally) but also at a larger, whole-organism scale (systemically). In general, the focal response represents the alterations driven by direct host-pathogen interactions at the infection sites where direct cell-cell (e.g., molecular patterns) interactions mediate the response; while the systemic response reflects overall modifications within the host as a result of the ongoing infection and is mainly associated with dynamic changes of circulating hemocytes and their secreted immune mediators.

Most of the previous investigations have solely focused on the systemic response of *M. mercenaria* against QPX during the infection events, where changes in cellular and humoral immune parameters (e.g., anti-QPX activity and lysozyme activity in clam plasma, hemocyte phagocytic activity, reactive oxygen species (ROS) production, etc.) as well as expression of a limited number of immune-related genes in tissues and circulating hemocytes were assessed [11, 14–16]. In contrast, no previous studies have focused on the characterization of clam focal response at the infection sites. Given the fact that QPX disease is usually focal with formation of well-delimited lesions, the study of clam immune responses at the infection site in the lesions *per se* is of specific value as it provides insights to better characterize cellular interactions between the hard clam and QPX upon their contact. In this framework, QPX disease in clams offer a unique opportunity to contrast focal and systemic responses against microbial diseases in invertebrates allowing for a more comprehensive understanding of defense strategies used by these animals to fend microbial attacks.

Our study aimed to characterize the gene regulation features of *M. mercenaria* during QPX infection by profiling the transcripts at the infection lesion and compare focal clam responses with systemic responses detected in healthy tissues from infected clams (in addition to a parallel comparison with tissues from healthy clams). This study allowed the identification of factors involved in the interactions with the parasite as well as molecular pathways activated by the host to neutralize QPX.

## Results and discussion

### Illumina sequencing and *de novo* transcriptome assembly

**Fig. 1:**
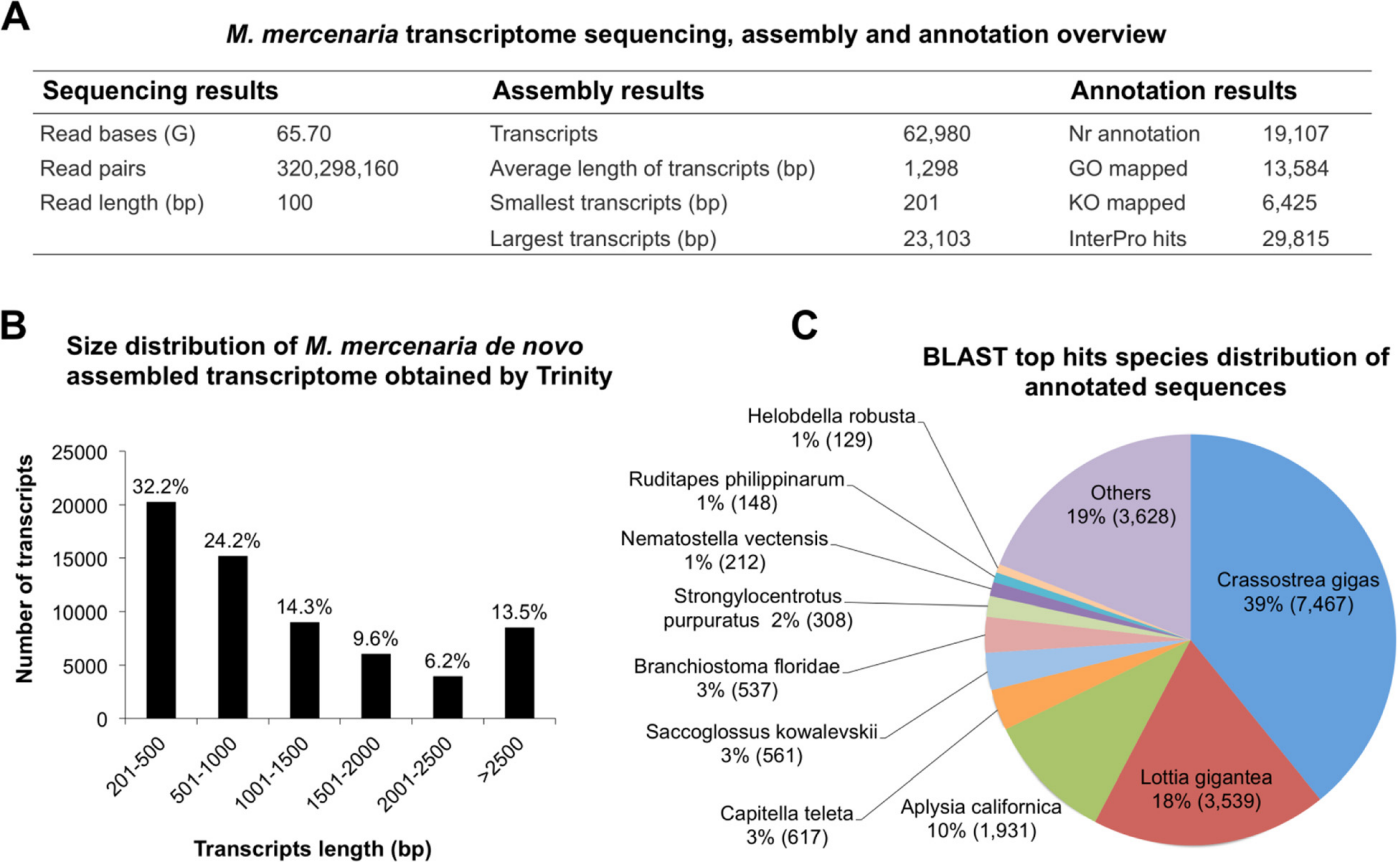
*M. mercenaria de novo* assembled transcriptome summary. **a** Transcriptome sequencing, assembly and annotation overview. **b** Assembled transcripts size distribution. **c** Distribution of the top 10 species with most homologues to *M. mercenaria.* Transcripts were searched using BLASTx against NCBI nr database with a cutoff value of E < 10E-03

The main objective of this study was to identify molecular features of *M. mercenaria* in response to QPX infection and to compare the immune-related pathways involved in the lesion-specific focal response with the whole-organism scale systemic response. The high-throughput Illumina RNA sequencing and *de novo* assembly employed in this investigation allowed the construction of the transcriptome in the absence of *M. mercenaria* genome information. A total of 640,596,320 of 100 bp raw reads were generated from the Illumina paired-end sequencing with about 27 to 48 Millions paired-end reads generated from each of the 9 sequenced libraries (Table 1, Fig. 1a). The short read sequences generated from this RNAseq project have been deposited at the NCBI short Read Archive database under the SRA accession number SRP068241. Trimming and filtering procedures yielded 606,021,407 clean reads that were used for the *de novo* assembly of consensus transcriptome based on all sequenced RNA libraries in order to maximize the diversity of transcripts. This allowed 90.61 to 92.20 % of the reads from the 9 libraries be used for the transcriptome assembly. A total of 62,980 transcripts ranging from 201 to 23,103 bp with average size of 1297.59 bp and median size of 835 bp were produced from the assembly after low FPKM and rare isoforms filtering. The size distribution of all the *de novo* assembled transcripts is shown in Fig. 1b. Once the transcriptome was constructed, the 9 libraries were individually remapped to the 62,980 transcripts and resulted with 85.27 to 89.05 % of reads remapping. Theses counting data were then used for DE analysis.

**Table 1:** RNA samples for RNA-seq libraries. Each pool is made with equal amounts of RNA from 3 individual clams. Pools A and B were derived from the same clams

	Library	Clams	Clam status	Tissue status	N paired-end reads
Nodule	A1	1, 2, 3	Diseased	Infection foci	30,491,569
	A2	4, 5, 6	Diseased	Infection foci	34,515,597
	A3	7, 8, 9	Diseased	Infection foci	46,861,893
Non-nodule	B1	1, 2, 3	Diseased	Non-lesion/Healthy	27,119,432
	B2	4, 5, 6	Diseased	Non-lesion/Healthy	28,254,720
	B3	7, 8, 9	Diseased	Non-lesion/Healthy	40,259,333
Healthy	C1	10, 11, 12	Healthy	Healthy	36,714,347
	C2	13, 14, 15	Healthy	Healthy	43,293,545
	C3	16, 17, 18	Healthy	Healthy	42,046,740

### Transcriptome functional annotation

The transcriptome annotation performed using Blast2GO returned a total of 19,107 transcripts (30.3 %) with significant BlastX homology matches to other sequences in NCBI nr database (E-value < 10E-03) (Fig. 1). Not surprisingly, the top 3 species that had the most similarity to *M. mercenaria* sequences were mollusks with available genomes and included the Pacific oyster, *Crassostrea gigas* (7,467), followed by the owl limpet *Lottia gigantea* (3,539) and the California sea slug *Aplysia californica* (1,931) (Fig. 1c). KEGG Orthology (KO) terms were assigned to 6,425 sequences and reference pathways were mapped to the KEGG database based on the assigned KO terms (Fig. 1a, Additional file 1). A total of 29,815 sequences were identified to match to at least one conserved protein domain in the InterPro database (Fig. 1a, Additional file 1).

**Fig. 2:**
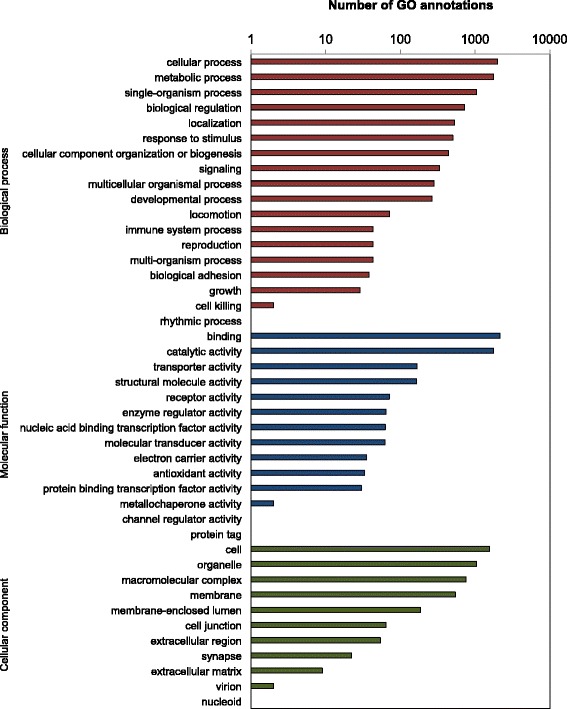
Gene Ontology (GO) annotations of the *M. mercenaria* transcriptome. GO terms were identified by Blast2GO and the results were summarized in three main GO categories: biological process (8,168 annotations), cellular component (4,600 annotations), molecular function (4,231 annotations) at level-2

Gene ontology (GO) assignments were used to classify functions of the predicted clam proteins. Based on sequence similarity (E-value of 10E-03), 13,584 sequences were assigned to at least one GO annotation (Fig. 1a, Additional file 1). As summarized in Fig. 2, a total of 8,168, 4,600 and 4,231 sequences were respectively categorized into the three main categories: biological process, cellular component, and molecular function at the second functional annotation level. The most dominant terms presented in the three categories are the “cellular process”, “metabolic process”, “binding”, “catalytic activity”, “cell”, and “organelle”. Very few transcripts were clustered into “rhythmic process”, “cell killing”, “protein tag”, “channel regulator activity”, “nucleoid” or “virion”. It is noticeable that a good fraction of transcripts were clustered into the immune-related categories of response to stimulus (503), immune system process (43) and biological adhesion (38). Those transcripts were of special interest given that they might be involved in the *M. mercenaria* defense and resistance toward QPX infection.

A significant portion (69.7 %) of *M. mercenaria* transcripts did not match any BlastX hit in NCBI nr database, in agreement with previous transcriptomic studies in mollusks [17–20]. Most of the unannotated transcripts may represent transcripts spanning untranslated mRNA regions, or transcripts containing only non-conserved protein domains [21, 22].

### Identification of differentially expressed transcripts

The generated transcriptome was used as a reference for downstream investigations of global gene expression in the three different tissues of interest (nodule, non-nodule and healthy) to identify genes associated with *M. mercenaria*’s focal and systemic immune response against QPX. A gene-isoform relationship was estimated using RSEM over Trinity output isoforms. Results showed that about 43 % (27,021) of all the transcripts had 1 isoform, 19 % (12,307) had 2 isoforms and 38 % (23,652) had 3 isoforms, suggesting extensive isoform diversity in *M. mercenaria* transcriptome. Transcript isoform variation could affect mRNA stability, localization and translation, as well as the production of protein variants that differ in localization or function [23].

**Fig. 3:**
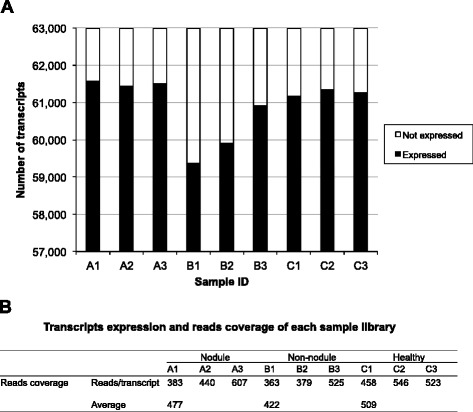
Number of transcripts expressed and reads coverage in each sample. **a** The X-axis indicates the sample (A1-A3: nodule; B1-B3: non-nodule; C1-C3: healthy, refer to Table 1 for more details). The Y-axis indicates the number of transcripts expressed in the samples. **b** Summary statistics of the reads coverage in each sample

By comparing the number of transcripts expressed in each sample, the contribution of specific samples to the analysis can be estimated. The highest number of expressed transcripts was found in the nodules of infected clams, which were closely followed by that found in the healthy clam samples (Fig. 3a). The lowest number of expressed transcripts came from non-nodule samples of QPX infected clams, with about 1,500 less transcripts expressed than the other two samples. Read coverage, which is critical in accurate determination of fold change, averaged 477, 422 and 509 reads per transcript for nodule, non-nodule and healthy tissue samples, respectively (Fig. 3b).

**Fig. 4:**
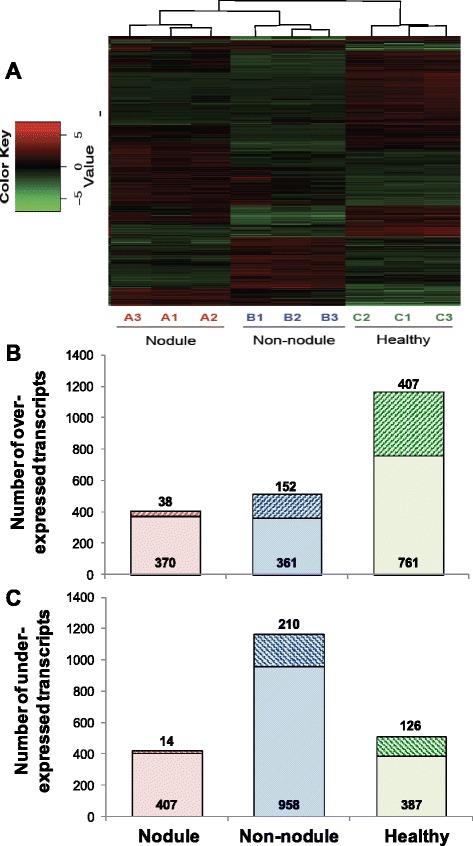
Heatmap (**a**) and number of differentially expressed transcripts (**b** and **c**) across all samples (FDR ≤0.001, and |log_2_ (fold change)| >2). Over-expressed transcripts are shown in red in A and are enumerated in B. Under-expressed transcripts are shown in green in A and are enumerated in C. Replicate biological samples are displayed in A (see Table 1 for more details). For B and C: the cross-shaded areas inside each bar represent the number of transcripts with higher (B) or lower (C) expression levels in a condition as compared to the other two (e.g., the expression levels of 38 focally over-expressed transcripts in B were higher in nodule than in non-nodule tissues and healthy clams)

Statistical analysis by DEseq identified 3,131 differentially expressed (DE) transcripts from the pair-wise comparisons (|log_2_ (fold change)| >2, adjusted *p*-value <0.001) between clam tissue samples (Fig. 4). In nodules, a total of 829 transcripts, including 408 over-expressed and 421 under-expressed transcripts were identified as compared to non-nodule samples of QPX infected clams. Compared to tissues from healthy clams, 1,591 DE transcripts were identified in nodules with 864 over-expressed and 727 under-expressed transcripts. Similarly, 1,681 DE transcripts were obtained from the comparison between healthy and non-nodule clam tissues, of which 513 and 1,168 were over- and under-expressed, respectively (Fig. 4, Additional file 3). A total of 1,694 of these DE transcripts had protein homologs found in NCBI nr database by Blastx searches (e-value <10E-03), which were further examined for their putative functions during *M. mercenaria* immune response toward QPX. Annotated transcripts were subsequently grouped into curated categories according to their biological functions based on the gene ontology (GO) terms and literature searches highlighting immune functions. DE transcript sets were further examined in reference to the assigned KO terms for the analysis of pathways regulation. Significantly enriched KEGG pathways were identified via the Fisher’s exact test (*P* < 0.01) [24].

Here we specifically focus on DE transcripts drawn from comparisons between “nodule vs. non-nodule” and “non-nodule vs. healthy” tissue samples, which were respectively considered to reflect the transcriptomic changes caused by “focal” and “systemic” immune response of *M. mercenaria* toward QPX infection, respectively. The overview of DE transcripts drawn from these two responses is presented in Fig. 4 and Additional files 2 and 3.

### Differential expression of immune related transcripts

#### Focal response

Clam focal response reflected the alterations caused by direct clam-QPX interaction at the infection site (Additional file 2). QPX nodules are inflammatory masses resulting from massive hemocytes infiltration and encapsulation of parasite cells [1, 2]. This process largely relies on the motility and adhesion properties of hemocytes, thus allowing these cells to migrate throughout the circulatory system and recruit to the infection site. Hemocytes can sense stimuli in host tissues through an array of cell surface receptors, and use these cues to adjust their behavior accordingly [25]. The activation of hemocytes requires the binding of specific ligands to the cell surface receptors, which subsequently initiate the transduction of extracellular signals into the cytoplasm via a variety of signaling pathways, thus inducing a series of hemocyte-mediated immune response such as phagocytosis, encapsulation (prominent response against QPX in clams), ROS production, as well as secretion of immune effectors and cytokines [26–28]. A collection of DE transcripts involved in these defense processes was identified during focal response, suggesting that strong and comprehensive host-pathogen interactions were taking places inside the QPX lesions (Table 2, Additional file 2). A large fraction of the DE transcripts of focal response was annotated as receptors or molecules with receptor activities, which putatively contribute to the host defense against QPX as (1) cell surface receptors expressed on hemocytes that mediate the recognition and phagocytosis or encapsulation of foreign entities through microbe associated molecular patterns (MAMP); (2) signaling receptors activating intracellular signaling cascades or (3) the soluble bridging molecules mediating the linkage between MAMPs and hemocytes [29]. Among those receptors, most are identified as pathogen pattern recognition receptors (PRRs), which include the C-type lectins (CTLs), the scavenger receptors (SRs) and the toll-like receptors (TLRs).

**Table 2:** Transcripts with annotated functions (GO terms) related to immune recognition, signaling and regulation that were differentially expressed during *M. mercenaria* focal response against QPX. Additional information on these transcripts is given in Additional file 2. “Inf” designates an infinite fold change calculated for focal response as the expression of that transcript in non-nodule tissue was equal to 0

Transcripts ID	Annotation	Regul-ation	Fold change	Function/GOs
Pathogen recognition receptors (PRRS)				
Scavenger receptors				
comp186077_c0_seq3	somatomedin-b and thrombospondin type-1 domain-containing (RPE spondin)	Up	71.3	F:scavenger receptor activity; P:immune response; F:polysaccharide binding
comp179365_c0_seq8	insulin-related peptide receptor	Up	11.5	F:scavenger receptor activity; P:transmembrane receptor protein tyrosine kinase signaling pathway
comp169098_c0_seq1	hemicentin-1	Up	10.6	F:scavenger receptor activity; P:cell adhesion
comp169486_c0_seq1	lysyl oxidase-like protein 2	Up	22.5	F:scavenger receptor activity; F:copper ion binding
comp192413_c0_seq18	mam domain-containing glycosylphosphatidylinositol anchor protein 1	Up	Inf	F:polysaccharide binding; F:scavenger receptor activity; P:immune response
C-type lectin (CTL)				
comp176879_c0_seq8	c-type mannose receptor 2	Up	15.8	F:binding; F:carbohydrate binding
comp190222_c0_seq3	perlucin-like protein	Up	49.1	F:binding; F:carbohydrate binding;
Toll-like receptors (TLRs)				
comp189381_c0_seq1	toll-like receptor 1	Up	16.5	F:protein binding
comp190056_c0_seq8	toll-like receptor e precursor	Down	Inf	P:signal transduction; F:protein binding
comp188195_c0_seq7	toll-like receptor g precursor	Up	Inf	F:protein binding; P:signal transduction
comp189381_c1_seq2	cell surface receptor tollo (toll 8)	Up	10.9	P:signal transduction; F:protein binding
Integrin				
comp189082_c0_seq13	integrin alpha 4	Up	Inf	P:cell-cell adhesion; C:integrin complex
comp189082_c0_seq24	integrin alpha 4	Up	16.2	P:cell-cell adhesion; C:integrin complex
comp185789_c0_seq4	integrin alpha-ps	Up	13.7	P:cell adhesion; C:integrin complex
comp184975_c0_seq10	integrin beta-1-like	Up	12.4	F:protein binding; F:receptor activity;
Low-density lipoprotein (LDL)				
comp181432_c3_seq2	LDL receptor-related protein 12	Up	12.1	-
comp187193_c1_seq4	LDL receptor-related protein 12	Up	Inf	F:protein binding
G-protein coupled receptor				
comp177186_c1_seq2	G-protein coupled receptor partial	Up	13.0	P:cell surface receptor signaling pathway; F:transmembrane signaling receptor activity
comp191316_c2_seq1	G-protein coupled receptor 64-like	Up	28.1	-
comp177657_c2_seq5	guanine nucleotide-binding protein subunit	Up	Inf	P:G-protein coupled receptor signaling pathway; F:signal transducer activity
comp187628_c0_seq44	substance-k (neurokinin) receptor	Up	9.1	P:G-protein coupled receptor signaling pathway; F:G-protein coupled receptor activity;
comp192446_c0_seq4	orexin receptor type 2	Up	12.4	P:G-protein coupled receptor signaling pathway;
Immune signaling and cell communication				
Phosphatase and kinases				
comp192546_c5_seq4	immunoglobulin i-set domain protein	Up	13.2	F:protein serine/threonine kinase activity
comp165267_c0_seq3	von willebrand factor type egf and pentraxin domain-containing protein	Up	11.0	F:protein tyrosine kinase activity
comp191437_c1_seq4	focal adhesion kinase 1	Up	19.7	F:signal transducer activity;
comp188649_c0_seq3	calcium calmodulin-dependent protein kinase	Up	9.3	F:calmodulin-dependent protein kinase activity
comp190317_c0_seq7	neuronal cell adhesion	Down	−13.3	F:rhodopsin kinase activity
Rho signaling				
comp189234_c1_seq21	rho gtpase-activating protein 15-like isoform x3	Up	18.1	P:signal transduction
comp185122_c1_seq7	rho gtpase-activating protein 24	Up	27.1	P:signal transduction
comp187736_c1_seq25	rho-related protein raca	Up	Inf	F:protein binding
comp188711_c0_seq2	rho-associated protein kinase 2	Up	Inf	P:intracellular signal transduction
Ubiquitin pathway				
comp190451_c2_seq9	e3 ubiquitin-protein ligase hectd1	Up	164.9	F:ubiquitin-protein ligase activity; F:metal ion binding
comp184512_c0_seq2	e3 ubiquitin-protein ligase march6	Up	15.0	F:zinc ion binding
comp192081_c0_seq1	e3 ubiquitin-protein ligase ubr3	Up	8.7	-
comp179031_c0_seq4	cop9 signalosome complex subunit 5	Up	Inf	F:protein binding
comp179031_c0_seq2	cop9 signalosome complex subunit 5	Up	Inf	F:protein binding
Wnt and Notch pathway				
comp185090_c0_seq10	tyrosine-protein kinase ryk	Up	26.3	P:Wnt receptor signaling pathway
comp187449_c0_seq2	fizzy-like protein	Up	13.0	F:protein binding
comp175460_c0_seq1	neurogenic locus notch	Up	11.7	P:Notch signaling pathway;
comp182793_c0_seq5	neurogenic locus notch protein	Up	9.6	P:G-protein coupled receptor signaling pathway
comp192565_c0_seq10	nicastrin-like protein	Up	12.6	P:protein processing
Calcium mediated signal transduction				
comp183265_c0_seq1	calmodulin 3b (phosphorylase delta)	Down	−11.6	F:calcium ion binding
comp191993_c0_seq4	EF-hand Ca-binding domain-containing protein 5	Down	−10.9	-
comp191855_c0_seq2	EF-hand Ca-binding domain-containing protein 6	Down	−15.9	-
Complement pathway				
comp165285_c0_seq7	macrophage-expressed gene 1	Up	11.5	-
Signal transducer				
comp182953_c0_seq4	signal recognition particle receptor subunit alpha	Up	13.8	F:signal recognition particle binding
comp182953_c0_seq5	signal recognition particle receptor subunit alpha	Up	73.0	F:signal recognition particle binding
comp171563_c0_seq4	gtp-binding nuclear protein	Up	Inf	P:small GTPase mediated signal transduction
comp188686_c0_seq19	neuralized pats1	Down	−758.9	P:small GTPase mediated signal transduction
comp189853_c0_seq1	unc5c-like protein	Down	−12.5	P:signal transduction
Cell death regulation				
Apoptosis process				
comp175357_c1_seq16	solute carrier family 25 member 38-like isoform 1	Down	−25.0	P:transmembrane transport
comp191590_c0_seq3	p53-induced protein with a death domain isoform	Down	−39.1	F:protein binding; P:signal transduction
comp191147_c0_seq62	inhibitor of apoptosis	Up	24.6	-
comp191147_c0_seq70	inhibitor of apoptosis	Up	Inf	F:metal ion binding; F:zinc ion binding;
comp191055_c2_seq4	programmed cell death protein 10	Up	367.1	-
comp190690_c2_seq9	cell death abnormality protein 1-like	Up	Inf	F:binding; F:zinc ion binding
comp186101_c3_seq4	3-hydroxy-3-methylglutaryl-coenzyme A reductase	Down	−10.8	P:positive regulation of apoptotic process; P:negative regulation of wound healing; P:oxidation-reduction process;
Tumor necrosis factor (TNF)				
comp182922_c0_seq5	TNF ligand superfamily member 10-like	Up	8.5	-
comp176786_c2_seq3	TNF-like protein	Down	−26.3	F:tumor necrosis factor receptor binding; P:immune response
	TNF ligand superfamily member 10-like	Up	8.5	-

The C-type mannose receptor-2 (MRC2) identified during the focal response (Table 2) is a member of the C-type lectins (CTLs) superfamily, a large group of Ca^2+^-dependent carbohydrate-binding proteins that play crucial roles in innate immunity. CTLs recognize pathogens and facilitate their phagocytosis [30, 31] or encapsulation [32–34]. MRCs are also key regulators of inflammatory responses and contribute to the removal of harmful inflammatory agents [35, 36]. The 16-fold over-expression of MRC2 during the focal response suggested that active hemocyte encapsulation and local inflammation was induced by QPX at the infection lesions, which is consistent with the results of histopathological observations [1]. Another over-expressed CTL member, the perlucin-like protein, has been previously shown to trigger immune response in Manila clams during microbial infection [37].

Scavenger receptors (SRs) were also among the strongly over-expressed transcripts in infection foci (Table 2). These included somatomedin-b and thrombospondin type-1 domain-containing (RPE spondin), insulin-related peptide receptor, hemicentin-1, lysyl oxidase-like protein 2 and mam domain-containing glycosylphosphatidylinositol anchor protein 1. SRs are structurally diverse PRRs that share the common function of recognizing oxidized or acetylated low-density lipoprotein (LDL) [38]. They contribute to innate immunity by recognizing MAMPs and mediating non-opsonic phagocytosis [29, 39]. They are extensively found on immune cells and are able to interact with both modified-host components and exogenous ligands, which makes SRs a key component in host defense, apoptosis, inflammation and lipoprotein homeostasis [40–42]. For example, scallop SRs bind not only to acetylated LDL but also to MAMP including lipopolysaccharides (LPS), peptidoglycans (PGN), mannan and zymosan particles [39]. The sea urchin genome encodes approximately 150 genes consisting of one or more scavenger receptor cysteine-rich (SRCR) domains [43], and the members of this gene family exhibit dynamic shifts in transcription after immune challenge [40, 44].

Our results also show an over-expression of TLR-1 and Toll-8/tollo in nodules (Table 2), which is in agreement with previous investigations showing up-regulation of TLRs in *M. mercenaria* mantle following QPX challenge [15]. TLRs are among the most ancient and conserved PRRs. They are expressed by immune cells and interact with a large variety of MAMPs. Bivalve TLRs have been characterized in the oyster *C. gigas* and the scallop *C. farreri* where they exhibited significant response to LPS stimulation [45, 46]. Transcriptional modulation of TLRs has also been reported in *Ruditapes philippinarum* and *Mya arenaria* following MAMPs stimulation and bacterial challenge [47, 48]. Interestingly, Toll-8 (Tollo) has been shown to participate in *Drosophila* epithelial immunity where it mediates host cells communication that subsequently activates systemic immune responses [49]. This suggests that the Toll pathway could be one of the crucial pivoting links that allow coordination between focal and systemic immune components during infection.

The QPX nodules are formed as the result of granulomatous inflammation, which is a chronic inflammatory reaction characterized by focal accumulation of activated immune cells to isolate the invading agent [50, 51]. The formation of granuloma requires local recruitment of hemocytes at the site of infection to execute extracellular defense processes around the invaders [50]. An array of transcripts associated with cell migration, adhesion and proliferation was regulated in nodules, including G-protein coupled receptors (GPCRs) and integrins families (Table 2). GPCRs regulate inflammatory response via binding to chemokines and chemoattractants, thus activating pathways mediating hemocyte migration and adhesion [52]. They also activate transcription factors in immune cells, thus modulating the synthesis and secretion of certain pro- or anti-inflammatory substances [53]. On the other hand, integrins represent a major group of cell adhesion mediators [54]. They not only modulate the cell-cell and cell-extracellular matrix adhesion, but also affect multiple signal transduction cascades regulating cell survival and proliferation [54]. Overexpression of GPCRs and integrins in nodules suggests their role in hemocytes adhesion and aggregation associated with the formation of granuloma [50, 55].

Several enzymes regulating ROS production were also over-expressed during focal response (Table 3). These included a dual oxidase, which is a key component mediating host-microbe interactions in mucosa [56, 57]. Dual oxidase regulates oxidative burst and ROS production in the gill muscosa of the shrimp *Marsupenaeus japonicus*, favoring shrimp survivorship during viral infections [58]. Interestingly, transcripts of dual oxidase were only expressed in nodules, suggesting this enzyme was induced upon direct clam-QPX interactions as a part of the mantle mucosa-related immune response. Other transcripts associated with oxidation-reduction processes also exhibited somewhat nodule-exclusive pattern, including the allene oxide synthase-lipoxygenase (AOSL), lysyl oxidase-like protein (LOXL), ww domain-containing oxidoreductase (WWOX), c-terminal binding protein (CtBP), isocitrate dehydrogenase (ICD) and methylenetetrahydrofolate reductase (MTHFR). These molecules are important for maintaining the redox homeostasis of extracellular environment as they are key regulators for oxi-reduction reactions. Over-expression of these transcripts in nodules suggests the need for the host to timely balance out excessive ROS and other toxic intermediates produced during interaction with QPX. In addition to redox-regulation, many of these molecules also take part in the immune modulation indirectly. For example, AOSL play a role in coral immunity by controlling the production of the inflammation regulator arachidonic acid during apoptosis [59], and LOXL acts both as a scavenger receptor and regulator for extracellular matrix remodeling that initiate hemocyte migration and tissue regeneration [60], while WWOX was shown to promote proliferation of immune cells through inhibition of their apoptosis [61, 62]. In addition, ICD, MTHFR and cytochrome p450 are major detoxification enzymes [63, 64]. In fact, immune cells and their secreted effectors require the proper redox state in the extracellular environments to exert their immune functions, which makes the maintenance of redox homeostasis essential for persistent and effective host defense [65, 66]. This is particularly true in the case of QPX disease where the neutralization of parasites depends on extracellular killing pathways [26].

**Table 3:** Transcripts with putative functions (GO terms) related to immune effectors that were differentially expressed during *M. mercenaria* focal response against QPX. Additional information on these transcripts is given in Additional file 2. “Inf” designates an infinite fold change calculated for focal response as the expression of that transcript in non-nodule tissue was equal to 0

Transcripts ID	Annotation	Regula-tion	Fold change	Function/GOs
Oxidation-reduction processes				
comp186178_c0_seq12	dual oxidase	Up	Inf	P:response to oxidative stress; P:oxidation-reduction process; F:peroxidase activity
comp185478_c0_seq4	allene oxide synthase-lipoxygenase protein	Up	10.0	P:oxidation-reduction process; F:oxidoreductase activity; F:metal ion binding;
comp186926_c0_seq7	c-terminal-binding protein	Up	Inf	P:oxidation-reduction process; F:NAD binding;
comp187462_c1_seq6	chorion peroxidase	Up	11.8	P:oxidation-reduction process; P:response to oxidative stress; F:peroxidase activity
comp157634_c0_seq8	cytochrome p450	Up	178.6	P:oxidation-reduction process; F:oxidoreductase activity; F:iron ion binding
comp183426_c1_seq2	dbh-like monooxygenase protein 1-like protein	Up	24.8	P:oxidation-reduction process; F:dopamine beta-monooxygenase activity; F:oxidoreductase activity
comp188723_c0_seq1	isocitrate dehydrogenase	Up	Inf	P:oxidation-reduction process; F:magnesium ion binding; F:NAD binding
comp169486_c0_seq1	lysyl oxidase-like protein 2	Up	22.5	F:oxidoreductase activity; P:oxidation-reduction process; F:scavenger receptor activity
comp192316_c0_seq4	methylenetetrahydrofolate reductase	Up	Inf	P:oxidation-reduction process; F:methylenetetrahydrofolate reductase (NADPH) activity;
comp189621_c0_seq4	procollagen-oxoglutarate 5-dioxygenase 3	Up	9.7	P:oxidation-reduction process; F:iron ion binding
comp185148_c0_seq6	ww domain-containing oxidoreductase	Up	Inf	F:oxidoreductase activity; P:metabolic process
Protease				
comp184786_c1_seq4	fur protein precursor	Up	Inf	F:serine-type endopeptidase activity
comp180950_c0_seq5	lysosomal protective protein precursor	Up	47.1	F:serine-type carboxypeptidase activity
comp180950_c0_seq2	lysosomal protective protein precursor	Up	26.9	F:serine-type carboxypeptidase activity
comp189961_c0_seq12	N-acetylated-alpha-linked acidic dipeptidase 2	Up	16.3	F:metallopeptidase activity;
comp178551_c1_seq1	membrane metallo-endopeptidase-like 1-like	Up	Inf	F:metalloendopeptidase activity;
comp184011_c0_seq4	blastula protease 10	Up	8.1	F:metalloendopeptidase activity;
comp191868_c1_seq1	matrix metalloproteinase-19	Up	59.1	-
comp174947_c0_seq3	isoaspartyl peptidase l-asparaginase-like	Up	Inf	F:hydrolase activity
comp183848_c0_seq3	kyphoscoliosis peptidase	Down	−8.7	P:microtubule-based movement
comp188831_c0_seq3	puromycin-sensitive aminopeptidase-like isoform	Down	−65.5	F:metallopeptidase activity;
comp191458_c3_seq5	aspartic protease with reverse transcriptase activity	Down	−245.4	F:aspartic-type endopeptidase activity;
Protease inhibitor				
comp189919_c1_seq4	alpha macroglobulin	Up	21.9	F:endopeptidase inhibitor activity
comp189919_c1_seq2	alpha macroglobulin	Up	12.4	F:endopeptidase inhibitor activity
comp181286_c4_seq1	thioester-containing protein	Up	10.2	F:endopeptidase inhibitor activity
comp191416_c1_seq2	thioester-containing protein-a	Up	14.8	F:endopeptidase inhibitor activity
comp191416_c1_seq1	thioester-containing protein-b	Up	9.5	F:endopeptidase inhibitor activity
comp192366_c0_seq1	thioester-containing protein-c	Up	32.5	F:endopeptidase inhibitor activity
comp191416_c0_seq5	thioester-containing protein-e	Up	12.9	F:endopeptidase inhibitor activity
comp191416_c0_seq4	thioester-containing protein-e	Up	22.4	F:endopeptidase inhibitor activity
Ion transporter and sequester				
comp190604_c1_seq1	ceruloplasmin precursor	Up	21.8	P:copper ion transport; P:cellular iron ion homeostasis; F:ferroxidase activity
comp182612_c1_seq1	ferric-chelate reductase 1	Up	16.7	Iron transfer
comp180332_c1_seq1	ferric-chelate reductase 1-like	Up	11.8	Iron transfer
comp177359_c0_seq1	selenium binding protein	Down	−11.0	P:protein transport; F:selenium binding
comp174164_c0_seq20	divalent metal transporter	Up	328.2	P:transport; F:transporter activity
Wound repair				
comp185425_c1_seq1	actin-related protein 2 3 complex subunit 5-like	Up	Inf	C:cytoskeleton; P:regulation of actin filament polymerization
comp93954_c0_seq1	extracellular matrix protein 2 isoform1	Up	25.9	F:protein binding
comp188753_c5_seq1	cartilage matrix protein	Up	25.8	F:chitin binding; P:chitin metabolic process; C:extracellular region
comp142858_c0_seq1	epidermal growth factor-like protein 8-like	Up	18.5	F:protein binding; F:calcium ion binding
comp192650_c1_seq4	multiple epidermal growth factor-like domains 6	Up	21.9	F:protein binding
comp186665_c0_seq1	thrombospondin- partial	Up	106.9	F:protein binding
comp186665_c0_seq4	thrombospondin- partial	Up	38.0	F:protein binding
comp184960_c0_seq4	septin-7-like isoform 8	Up	Inf	P:cell cycle; F:GTP binding; C:septin complex
comp184960_c0_seq42	septin-7-like isoform 6	Up	8.9	-

Apoptosis is an essential host mechanism to effectively remove damaged and infected cells without causing inflammatory destructions to surrounding tissues [67, 68]. Interestingly, apoptosis seems to be largely inhibited during *M. mercenaria* focal response, as shown by the under-expression of pro-apoptosis transcripts (Table 2). For example, the tumor necrosis factor (TNF)-like protein and 3-hydroxy-3-methylglutaryl-coenzyme A (HMG-CoA) reductase-like protein were significantly under-expressed in nodules. Similarly, the pro-apoptotic p53-induced protein and solute carrier family 25 member protein were also under-expressed in nodules. Meanwhile, inhibitor of apoptosis protein (IAP) was over-expressed in nodules. IAPs regulate immune cell expansion and survival in highly inflammatory environments in mammals [69] and they may share similar function in clams by preventing hemocytes from death during interaction with QPX. In fact, ROS production during parasite killing may trigger apoptotic cell death in molluscs [67], and proper control of apoptosis mechanisms is required to maintain cellular homeostasis during immune response. This suspected inhibition of host apoptosis is supported by the above-mentioned over-expression of integrins, as these were shown to protect cells from apoptosis and induce anti-apoptotic pathways during cell adhesion and spreading in the snails *Lymnaea stagnalis* [70] and *B. glabrata* [71].

Infection and tissue injury trigger host immune responses via immune signaling pathways [72], by activating transcription factors and initiating the production of immune effectors and regulators. Immune signaling pathways identified in mollusks include Toll, MAPK/JNK and JAK/STAT signaling pathways [27, 47, 73, 74]. During *M. mercenaria* focal response to QPX, a variety of transcripts encoding kinases and phosphatases were over-expressed (Table 2), suggesting the involvement of MAPK and other kinase-mediated cascades in regulating the focal inflammatory response [75], whereas the under-expression of EF-hand domain containing protein and calmodulin may indicate the suppression of calcium-regulated pathways [76]. Overexpression of E3 ubiquitin-protein ligase and its upstream regulator COP9 signalosome suggests the activation of the damage surveillance ubiquitin/proteasome pathway [77–79]. In parallel, the over-expression of rho GTPase and rho kinase suggests the induction of anti-apoptotic Rho-mediated signaling pathway [80, 81] and reinforce the idea that apoptotic inhibition is extensively initiated by *M. mercenaria* to help fight QPX. However, a very limited number of DE transcripts was detected in relation to those conventional signaling pathways of innate immunity, such as the complement pathway and the Toll/TLR pathway. Only 1 transcript encoding macrophage-expressed gene protein 1 (MPEG1), a putative member of the complement pathway [82, 83], was differentially expressed in QPX nodules. As for the Toll/TLR pathway, only a few receptors were identified (Table 2) but none of their downstream components.

Interestingly, components of the Notch and the Wnt signaling pathways were over-expressed during focal response (Table 2). These included two putative Notch family members, the neurogenic locus Notch protein and the mediator protein nicastrin, and the tyrosin-protein kinase RYK which belongs to the Wnt pathway. Wnt signaling pathway regulates many cellular immune processes and is evolutionarily conserved across taxa [84, 85]. Wnt signaling has been intensively exploited for its regulatory functions during wound healing and tissue regeneration [86, 87], so its over-expression may be related to wound healing to repair damage resulting from tissue digestion by the parasite or tissue necrosis. The Notch signaling network was scarcely explored in bivalves even though it has been reported to be associated with several aspects of immune response in mammals [88], especially in regulating granulomatous reactions to foreign bodies [89]. As an evolutionarily conserved pathway involved in modulating the intercellular signaling, the Notch pathway presumably shares an equally important role in *M. mercenaria* by modulating the formation of granuloma. Notch signaling triggers macrophage expression of genes involved in pro-inflammatory responses [90], but can suppress inflammation responses triggered by canonical TLR cascade [91], in agreement with our observations. These tightly regulated mechanisms ensure tailored immune responses against different pathogens and are crucial for the host to achieve high immune efficiency while avoiding excessive immune activation and self-inflicted damages.

Several proteases (A.K.A proteinases, peptidases) were also differentially regulated during *M. mercenaria* focal response against QPX. These proteases mostly belong to the serine and metallo protease families and were generally over-expressed in QPX nodules (Table 3). Proteases serve as key immune modulators partially through their ability to digest and remodel the extracellular matrix and tissues associated with hemocyte activation [92]. Commonly associated with lysosomes and granules of inflammatory cells, serine proteases participate in immune regulation either directly by degradation of pathogens or indirectly through activation of cell surface receptors and signal molecules [92–94]. The function of metalloproteases in immune regulation is even more diverse, acting as immune effectors, signal transducers, and mediators of immune cell development and migration [95]. Metalloproteases are also known to be involved in many pro-inflammatory pathways, particularly in the Notch pathway where they act as a type of downstream element to Notch [96].

At the same time, immune effectors with known universal protease inhibitor activities, such as alpha2-macroglobulin (α 2 M) and thioester-containing protein (TEP, a subfamily of α 2 M), were also collectively overexpressed in QPX nodules (Table 3). The α 2 M superfamily inhibits peptidases of diverse origins [97, 98]. The simultaneous over-expression of proteases and protease inhibitors may reflect a finely adjusted defense response of *M. mercenaria* to maintain homeostasis and regulate self- and pathogen-derived proteases, as shown in other host-pathogen systems [92, 99], including bivalves [100]. Proteases have been identified as major virulence factors of QPX [101, 102], and are thought to degrade host proteinaceous immune effectors and hydrolyze host tissues to fulfill nutritional requirements. Therefore, inhibition of pathogen proteases contribute to host protection, and was shown to represent a determinant factor for resistance against infectious diseases in bivalves [103–106]. In parallel, TEPs have been extensively studied in mollusk immunity [99, 107], and beside their function as protease inhibitors, they also play a role as PRRs or opsonins to facilitate microbial phagocytosis and encapsulation. Consistent over-expression of TEPs was noted in this study in agreement with findings following experimental infection with QPX [15]. These results support a critical role of TEPs in clam immune response against QPX either via the protease-inhibitor activity of these proteins, or by mediating parasite encapsulation, or both.

In addition, several metal ion transporters were over-expressed in nodules (Table 3), including the putative copper ion binding protein ceruloplasmin precursor, the transferrin enzyme ferric-chelate reductase and the divalent metal transporter (A.K.A natural resistance-associated macrophage protein; 328 fold increase). These molecules contribute to host defense by controlling the supply of essential micronutrients in the vicinity of infection sites thus reducing parasite survival [108, 109] and favoring the production of antimicrobial factors [110]. It is noteworthy to point out that some of the focally over-expressed transcripts might be partly driven by the dramatic increase of hemocyte proportion within tissues in the vicinity of infection foci as compared to the surrounding host tissues [26, 111]. This would be especially the case for transcripts known to be highly expressed in hemocytes, such as the cell surface PRRs, secreted humoral immune effectors, cell signal transducers and enzymes associated with ROS production.

#### Systemic response

Significant transcriptomic regulations were observed during *M. mercenaria* systemic immune response against QPX, with a total of 1,681 DE transcripts, which is about two times the number of focal DE transcripts (829). However, only about one third of the DE transcripts (513) were over-expressed in response to QPX infection, the larger remaining part (1,168) represented significantly under-expressed transcripts (Fig. 4, Additional file 3), possibly due to the chronic stress imposed by the infection. Transcriptome-wide depression has been demonstrated in many marine invertebrates as the result of pathogenic or environmental stress [18, 59, 112–114]. A considerable number of systemically under-expressed transcripts was related to metabolism and biomineralization, which could be the result of host resource allocation during on-going infection. The chronic inflammation induced by infection likely created extra energy demands, which require resources being allocated from other physiological processes, such as growth and reproduction, to immune processes which is critical for the survivorship of the host. In fact, slow growth and lower tissue conditions are frequently observed in QPX-infected *M. mercenaria* [2], and similar energy trade-offs existed between immune defense and other energy expenditure pathways [115–118]. Interestingly, a suite of transcripts over-expressed during focal response was significantly under-expressed in non-nodule tissues as compared to healthy clams (Additional file 3), which included several immune effectors and mediators associated with nodule formation and focal inflammation (e.g., integrins, notch proteins and peroxidases). In fact, maintaining high levels of these focally-induced molecules could be costly and dangerous as some are toxic to both the parasite and the host, so their production must be restrained within areas where they can directly exert defense function, and reduced outside the infection foci to minimize risks of undesirable effects on the host [116, 117].

On the other hand, systemically over-expressed transcripts included stress proteins and other soluble immune factors such as lysozyme (c-type lysozyme 2), lectins (C1q domain containing protein, macrophage mannose receptor 1, low affinity immunoglobulin epsilon fc receptor), AMP (hemocyte defensin), proteases (cathepsin K, calpain 11, isoaspartyl peptidase/L-asparaginase, ASRGL, counting factor associated protein d) and ferric-chelate reductase (Table 4, Additional file 3). Over-expression of host stress proteins, such as heat shock proteins (HSP 70, HSP 90) and universal stress protein (USP) was also noted, in agreement with observations made during infection in other bivalve species [119–121]. Increased levels of stress proteins provide host cells with protection against incorrect protein folding caused by infection, inflammation, oxidative stress and other destructive events [122, 123]. The systemic over-expression of soluble immune effectors (e.g., humoral proteins) may help maintaining comparatively high immune capacity to prevent the spread of QPX (or secondary pathogens) throughout the host. In addition, transcripts of anti-apoptotic factors (IL17, deoxyguanosine, baculoviral map repeat-containing proteins) were also over-expressed during the systemic response, indicating that anti-apoptotic processes noted during focal response are not limited to the infection foci.

**Table 4:** Selected transcripts with annotated functions (GO terms) related to immune response that were over-expressed during *M. mercenaria* systemic response against QPX. Additional information on these transcripts is given in Additional file 3. “Inf” designates an infinite fold change calculated for focal response as the expression of that transcript in non-nodule tissue was equal to 0

Transcripts ID	Annotation	Regul-ation	Fold change	Function/GOs
Stress protein				
comp39934_c1_seq1	heat shock 70 kda protein	Up	Inf	P:response to stress
comp181704_c3_seq49	heat shock 70 kda protein 12b	Up	452.9	-
comp68505_c0_seq1	heat shock protein 70	Up	37.8	P:response to stress
comp38810_c0_seq1	hsp90 family member	Up	64.7	P:response to stress
comp192296_c2_seq3	usp-like protein isoform 2	Up	46.2	P:response to stress
Immune effectors				
comp192209_c0_seq3	hemocyte defensin partial	Up	173.5	P:defense response
comp186386_c5_seq2	c-type lysozyme 2	Up	20.0	-
comp186386_c5_seq1	c-type lysozyme 2	Up	Inf	-
comp164821_c0_seq2	c1q domain containing protein	Up	8.3	Lectin
comp164821_c0_seq3	c1q domain containing protein	Up	11.9	Lectin
comp190576_c0_seq27	low affinity immunoglobulin epsilon fc receptor	Up	134.1	F:carbohydrate binding; Lectin
comp190576_c0_seq26	low affinity immunoglobulin epsilon fc receptor	Up	201.6	F:carbohydrate binding; Lectin
comp191987_c0_seq10	macrophage mannose receptor 1-like	Up	7.8	F:carbohydrate binding; Lectin
comp189507_c1_seq9	ferric-chelate reductase 1	Up	10.7	Iron transport
Peptidases				
comp156268_c1_seq1	cathepsin k-like	Up	9.5	-
comp184090_c0_seq6	calpain 11-like	Up	10.1	-
comp189096_c0_seq4	counting factor associated protein d-like	Up	36.1	F:cysteine-type peptidase activity
comp176418_c0_seq1	isoaspartyl peptidase	Up	8.0	F:hydrolase activity
Anti-apoptotic factors				
comp178822_c0_seq1	interleukin IL17-like	Up	11.6	P:inflammatory response; F:cytokine activity
comp176786_c2_seq3	deoxyguanosine mitochondrial	Up	29.5	F:tumor necrosis factor receptor binding; P:immune response;
comp192603_c1_seq8	baculoviral iap repeat-containing protein 7-a	Up	10.3	P:negative regulation of peptidase activity
comp192603_c1_seq7	baculoviral iap repeat-containing protein 7-a	Up	14.3	P:negative regulation of peptidase activity
comp191786_c0_seq3	baculoviral iap repeat-containing protein partial	Up	8.9	-

#### Pathway alterations during *M. mercenaria*’s response to QPX

**Fig. 5:**
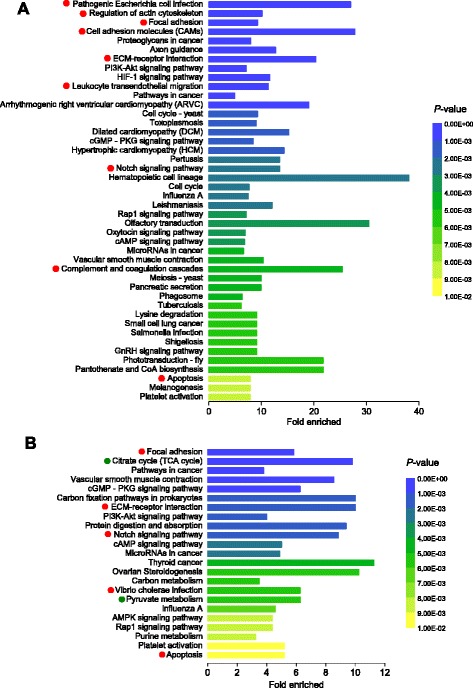
Significantly enriched KEGG pathways in *M. mercenaria* derived from the differentially expressed (DE) genes during focal (**a**) and systemic (**b**) response against QPX. The KEGG pathways having significant enrichment (*P* < 0.01) are presented, and the bar shows the x-fold enrichment of each KEGG pathway. Pathways involved in immune response are marked with red dots while metabolism-related pathways are marked with green dots

Transcriptomic alterations during both focal and systemic response discussed above were also highlighted in the pathway enrichment analysis of the DE transcripts. This analysis aims at extracting an overview of phenotypic changes on the underlying functional level, to reduce the complexity of biological information given by the long lists of DE genes/transcripts [124]. The KEGG pathways of focal adhesion (04510), ECM-receptor interaction (04512), Notch signaling pathway (04330) and apoptosis (0421) were significantly over-represented during both focal and systemic response (Fig. 5), even though fold enrichment were generally higher during the focal response. Other immune-related pathways particularly enriched during the focal response included regulation of actin cytoskeleton (04810), cell adhesion molecules (CAMs, 04514), the leukocyte transendothelial migration (04670), complement and coagulation cascade (04610) and Wnt signaling pathway (04310). These pathways are critically involved in the immune cells activation during migration, attachment and parasite encapsulation, which serve as the underlying mechanisms for nodule formation and QPX killing. On the other hand, basic metabolic pathways such as the citrate cycle (TCA) and pyruvate metabolism were specially enriched during the systemic response. These alterations were largely in accordance with the under-expression of metabolism-associated DE transcripts in infected tissue compared to the healthy tissue (Fig. 4), possibly reflecting changes in the energy allocation strategy during infection as discussed above.

**Fig. 6:**
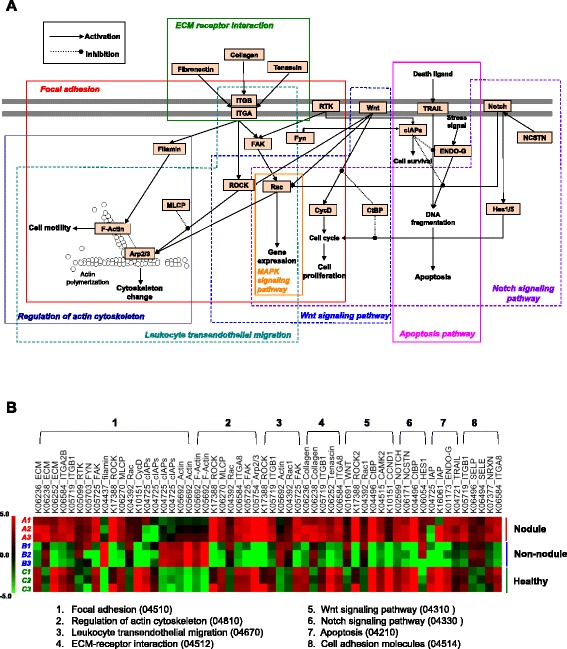
Overview of immune-related enriched pathways of differentially expressed (DE) transcripts during *M. mercenaria* response to QPX. **a** Schematic diagram of enriched pathways and their interactions. Only pathway components encoded by DE transcripts (shown in text boxes) are presented within each enriched KEGG pathway (framed in orthogonal polygons). Arrows display possible interactions (e.g., activation, inhibition) between pathway components. **b** Overview of DE transcripts expression associated with enriched pathways in nodule, non-nodule and healthy *M. mercenaria* tissues. The red and green heatmap values indicate log_2_ fold of relative expression levels for individual transcripts. Arp2/3: actin related protein 2/3 complex; CtBP: C-terminal binding protein; CAMK2: calcium/calmodulin-dependent protein kinase (CaM kinase) II; CycD: cyclin D1(CCND1); ECM: von Willebrand factor; ENDO-G: endonuclease G; FAK: focal adhesion kinase; FYN: tyrosine-protein kinase; HES1: hairy and enhancer of split 1; IAP: baculoviral IAP repeat-containing protein, cIAPs; ITGA: intergrin alpha; ITGB: intergrin beta; MLCP: serine/threonine-protein phosphatase PP1 catalytic subunit; NRXN: neurexin; NCSTN: nicastrin; Rac: Ras-related C3 botulinum toxin substrate; ROCK: Rho-associated protein kinase; RTK: proto-oncogene tyrosine-protein kinase; SELE: selectin, endothelial cell; SELP: selectin, platelet; TRAIL: tumor necrosis factor ligand superfamily member 10); WNT: wingless-type MMTV integration site family

Interdependence of KEGG pathways widely exist and most of these are interrelated with each other via shared components, forming a signaling network to allow for pathway crosstalk. To investigate these interactions, we extracted the DE transcripts shared by multiple enriched pathways and constructed a sketch of the hypothetical pathways network that are significantly altered by QPX infection (Fig. 6). In this framework, *M. mercenaria* response to QPX infection was initiated upon the sensing of danger signals via cell membrane receptors. The signals subsequently transmitted down through the MAPK, Wnt and Notch pathways and triggered the production of a series of host defense factors as the end results. In parallel, activation of pathways regulating actin cytoskeleton and leukocyte transendothelial migration facilitated the recruitment of hemocytes to the infection area to build a barrier of cellular defense against the parasite. Recruited hemocytes then attached to and encapsulated QPX cells as suggested by the modulation of focal adhesion and ECM receptor interaction pathways. These cellular activities were performed under a tight regulation of the apoptosis pathway to determine cell fates, resulting in either the survival or death of *M. mercenaria* cells.

#### Distinctive transcriptomic pattern of healthy clams

A suite of transcripts (407, Fig. 4, Additional file 4 and Table 5) exhibited higher transcription levels in healthy clams as compared to diseased clams (considering both nodule and non-nodule tissues). A considerable fraction of these transcripts were related to metabolic processes, nucleic acids binding and transcriptional regulation. The over-expressed immune-related transcripts identified in healthy clams are of particular interest as they may be involved in *M. mercenaria* resistance towards QPX. For example, an antimicrobial protein (aplysianin A [125, 126]) was exclusively identified in healthy clams with almost no detection in diseased clams. In addition, the highest expression levels of a serine protease inhibitor were also observed in healthy clams. A serine protease inhibitor has been linked to oyster (*Crassostrea virginica*) resistance against the protozoan parasite *Perkinsus marinus* [103–106, 127]. Therefore, the high expression of the serine protease inhibitor in healthy clams supports its involvement in *M. mercenaria*’s resistance against QPX, likely by inhibiting the activity of parasite proteases. Moreover, a pathogen recognition protein (c-type lectin domain family 10 member A-like) was significantly higher in healthy clams as compared to diseased animals, which may also contribute to clam resistance against QPX by promoting microbial recognition and encapsulation [128]. Previous studies demonstrated that clam genetic background affects *M. mercenaria* resistance toward QPX [4, 129, 130], therefore immune-related transcripts specifically associated with healthy clams represent excellent candidates as molecular markers for further research on screening and breeding QPX-resistant *M. mercenaria* strains.

**Table 5:** Selected transcripts with annotated functions (GO terms) related to immune response that were differentially expressed in naïve *M. mercenaria* as compared to QPX infected individuals. Additional information on these transcripts is given in Additional file 4. “Inf” designates an infinite fold change calculated for focal response as the expression of that transcript in non-nodule tissue was equal to 0

Transcripts ID	Annotation	Fold change	Function/GOs
Over-expressed			
comp168250_c0_seq2	macrophage expressed protein	84.0	-
comp160023_c0_seq1	serine protease inhibitor 1	18.1	F:protein binding
comp117137_c0_seq1	proline-rich transmembrane protein 1	148.6	P:response to biotic stimulus
comp134883_c0_seq1	c-type lectin domain family 10 member a-like	121.4	F:carbohydrate binding
comp180146_c3_seq31	aplysianin a precursor	Inf	P:defense response; F:oxidoreductase activity
comp179365_c0_seq7	insulin-related peptide receptor	20.0	F:scavenger receptor activity
comp187368_c2_seq9	apoptosis 1 inhibitor	16.2	P:apoptotic process;
comp193015_c0_seq1	baculoviral iap repeat-containing protein 4	391.0	P:negative regulation of apoptotic process; F:ubiquitin-protein ligase activity
Under-expressed			
comp39934_c1_seq1	heat shock 70 kda protein	Inf	P:response to stress
comp192296_c2_seq3	usp-like protein isoform 2	−46.2	P:response to stress
comp169961_c0_seq2	cytochrome p450 1a1	−75.1	-
comp176418_c0_seq1	isoaspartyl peptidase l-asparaginase-like	−8.0	F:hydrolase activity
comp191987_c0_seq10	macrophage mannose receptor 1-like	−7.8	F:carbohydrate binding
comp186563_c0_seq7	bile salt-activated lipase-like	−9.8	F:hydrolase activity

On the other hand, a subset of transcripts (126, Fig. 4 and Table 5) exhibited lowest expression levels in healthy clams. These include stress protein HSP 70 and USP, as well as the detoxification molecule cytochrome p450, which together highlight the stress experienced by clams as the result of QPX infection [63, 131–135]. In addition, other immune related transcripts including a protease (isoaspartyl peptidase/L-asparaginase, ASRGL), a protease inhibitor (GTP-binding protein yptV4), a C-type lectin (MRC1), and molecules involved in tissue regeneration and cell signaling, were also under-expressed in healthy clams as compared to infected individuals, suggesting their role in fighting the infection.

## Conclusions

This is one of the first studies contrasting focal and systemic immune responses to infections in invertebrates using high-throughput sequencing. Resulting transcriptome represents a significant addition to the so far limited public genomic information available for *M. mercenaria*. The transcriptomic profiles of healthy and infected clams reflected complex interactions between the host immune system and the pathogen leading to molecular changes at both the infection foci and the systemic level. In general, the systemic responses of *M. mercenaria* reflected prevailing transcriptomic suppression accompanied with a contrasting over-expression of stress proteins and soluble antimicrobial effectors; whereas the focal response highlighted cell-cell interactions between hemocytes and the parasite that typically result in local inflammation, extracellular degradation, encapsulation, granuloma formation, and wound repair. What needs to be kept in mind is that the regulation of these genes can be the result of either an effective immune response or a symptom of a future death. In fact, several apoptotic and anti-apoptotic genes were regulated. This highlights a fine adjustment of *M. mercenaria* defense mechanisms to precisely adapt to the infection development (e.g., through the modulation of energy allocation, apoptotic and anti-apoptotic processes and mobilization of different signaling pathways). In addition, the identification of immune-related transcripts that were particularly associated with healthy clams offered new perspectives on the molecular features putatively involved in clam resistance against QPX.

## Methods

### Clam tissue and RNA samples preparation

Adult hard clams (54 ± 5 mm in length, mean ± standard deviation) were collected from a QPX enzootic area in Massachusetts. Clams were shucked and grossly examined for the presence of nodules along the mantle rim. Nodule tissues were dissected and a small piece of each nodule was microscopically examined (fresh mount) for the identification of QPX cells. Aliquot tissues from positive nodules were submitted to RNA extraction. Meanwhile, a piece of seemingly healthy tissue that is anatomically symmetrical to the nodule was collected from the same clam (e.g., “healthy” tissue from a diseased clam) and divided into 2 aliquots, with the first aliquot used for QPX detection by qPCR [10] and the second used for RNA extraction. Mantle tissues were also collected from “seemingly” healthy clams (no visible nodules) and used for QPX detection and RNA extraction. Following confirmation of disease status by qPCR, the samples were divided into the following 3 categories: (1) infected tissue from a diseased clam, (2) “healthy”(non-nodule) tissue from a diseased clam, and (3) “healthy” tissue from a healthy clam. Total RNA was individually extracted using TRIzol ® Reagent (Invitrogen, Carlsbad, CA, USA). Further RNA clean-up and on-column DNase digestion were performed with RNeasy Mini Kit (Qiagen Ltd., Crawley, UK) according to the manufacturer’s guideline. RNA quantity and quality were analyzed on Nanodrop ND-1000 (Thermo Scientific, Wilmington, USA). Only RNA samples with absorption ratios of A260/A280 close to 2.0 were used for RNA-seq analysis. A total amount of 3 μg RNAs per sample was used and pooled into a total of 3 pools (3 clams per pool) representing each tissue category (9 pools in all). The pooling strategy is shown in Table 1.

### RNA sequencing, *de novo* assembly and annotation

The sequencing of each pooled RNA sample as a paired end (PE) reads library (100 pb) was performed on Illumina HiSeq 2000 platform at the McGill University and Genome Quebec Innovation Center (Montreal, Canada), producing from 27.1 up to 46.8 millions of PE reads per sample (Table 1). Raw reads were filtered and trimmed according to length and quality score (min length 60 nt, end trimming quality 25, min quality filtering: 20 on 75 % of the read length) using the FASTX-Toolkit software v 0.0.13 (http://hannonlab.cshl.edu/fastx_toolkit/). rRNA cleaning was performed using the riboPicker software v 4.0.3 (http://ribopicker.sourceforge.net) [136] against SILVA database v111. And finally pair retrieval was performed using a homemade python script accessible through GitHub (https://github.com/ppericard/bioinfo-toolkit). High quality filtered sequence reads from all libraries were combined and subsequently used for de novo assembly. Assembly was based on the de Bruijn graph assembler Trinity (http://trinityrnaseq.sourceforge.net/) [137] using the default parameters. Assembly quality was controlled by re-mapping raw reads back to the transcripts using bowtie 2 and RSEM within Trinity package scripts. The assembled transcripts with sequence length longer than 200 bp, re-mapping FPKM (fragments per kilobase of transcript per million mapped reads) greater than 1 and isoform discovery level greater than 1 % were then considered for the following annotation and transcript abundance quantitation. Annotation of this *de novo* assembled transcriptome was performed using Blast2GO (http://www.blast2go.com) with a semi-automated functional annotation based on sequence homology search. Putative gene identities was obtained by Blastx search against National Center for Biotechnology Information (NCBI) non-redundant sequences (nr) database with the E-value threshold setting at 1E-03. Putative gene functions were predicted by sequence similarity search against Gene Ontology (GO, http://www.geneontology.org/) database and assigning GO annotation terms to each mapped transcript. Protein domain search and enzyme annotation were also performed using InterPro scan and the Kyoto Encyclopedia of Genes and Genomes (KEGG). KEGG Orthology (KO) terms and KEGG pathways were also assigned to the assembled sequences using the online KEGG Automatic Annotation Server (KAAS, http://www.genome.jp/kegg/kaas/) using bi-directional best-hit methods [138]. This server provides KO annotation and pathway mapping.

### Differential gene expression analysis

The consensus transcriptome generated in the previous steps was used as a reference for transcript abundance analysis. Reads from each single library were separately mapped to this reference transcriptome using the “RNA-Seq by Expectation-Maximization (RSEM)” method that is bundled within the Trinity package. The expression level of each transcript was determined as the total mapped reads count. The differences in gene expression between clam tissue samples (nodule, non-nodule and healthy tissues) were estimated using the DESeq Bioconductor package (https://github.com/Bioconductor-mirror/SESeq/tree/release-3.2) [139] in R statistical software (R Development Core Team, 2010; http://www.R-project.org). The threshold for defining significant differentially expressed (DE) transcripts between two different conditions (3 replications in each condition) was set as adjusted *p*-value smaller than 0.001 and absolute log_2_ (fold change) values greater than 2. Expression patterns of DE transcripts were also analyzed by a K-means clustering method using Euclidean distance based on expression levels over all input samples. For further analysis, only those DE transcripts with annotation were considered as candidates of interest and were subsequently divided into over- and under-expressed groups.

## Availability of supporting data

The data sets supporting the results of this study are available at the NCBI short Read Archive database under the SRA accession number SRP068241.

## Additional files

Additional file 1: Sequence summary of *M. mercenaria de novo* assembled transcriptome. This is a Microsoft Excel worksheet that contains descriptions and annotation information of individual transcript sequences. (XLSX 6600 kb) Additional file 2: Differentially expressed (DE) sequences associated with *M. mercenaria* focal response against QPX. This file is a Microsoft Excel worksheet that contains separate lists of over- and under-expressed transcripts with their expression levels and fold change values given. (XLSX 143 kb) Additional file 3: Differentially expressed (DE) sequences associated with *M. mercenaria* systemic response against QPX. This file is a Microsoft Excel worksheet that contains separate lists of over- and under-expressed transcripts with their expression levels and fold change values given. (XLSX 327 kb) Additional file 4: Transcripts that were differentially expressed in naïve *M. mercenaria* as compared to QPX infected individuals. This file is a Microsoft Excel worksheet that contains separate lists of over- and under-expressed transcripts with their expression levels and fold change values given. (XLSX 128 kb)
